# Which Vaccine? The Cost of Religious Freedom in Vaccination Policy

**DOI:** 10.1007/s11673-021-10148-6

**Published:** 2021-12-23

**Authors:** Alberto Giubilini, Julian Savulescu, Dominic Wilkinson

**Affiliations:** 1 Oxford Uehiro Centre for Practical Ethics, University of Oxford, Oxford, UK; 2 Visiting Professorial Fellow in Biomedical Ethics, Murdoch Childrens Research Institute, Melbourne, VIC Australia; 3 Distinguished Visiting International Professorship in Law, University of Melbourne, Melbourne, VIC Australia

**Keywords:** Vaccination ethics, COVID-19, Pandemic ethics, Religious freedom

## Abstract

We discuss whether and under what conditions people should be allowed to choose which COVID-19 vaccine to receive on the basis of personal ethical views. The problem arises primarily with regard to some religious groups’ concerns about the connection between certain COVID-19 vaccines and abortion. Vaccines currently approved in Western countries make use of foetal cell lines obtained from aborted foetuses either at the testing stage (Pfizer/BioNTech and Moderna vaccines) or at the development stage (Oxford/AstraZeneca vaccine). The Catholic Church’s position is that, if there are alternatives, Catholic people have a moral obligation to request the vaccine whose link with abortion is more remote, which at present means that they should refuse the Oxford/AstraZeneca vaccine. We argue that any consideration regarding free choice of the vaccine should apply to religious and non-religious claims alike, in order to avoid religion-based discrimination. However, we also argue that, in a context of limited availability, considering the significant differences in costs and effectiveness profile of the vaccines available, people should only be allowed to choose the preferred vaccine if: 1) this does not risk compromising vaccination strategies; and 2) they internalize any additional cost that their choice might entail. The State should only subsidize the vaccine that is more cost-effective for any demographic group from the point of view of public health strategies.

## Introduction

Various COVID-19 vaccines have been or are about to be approved for use in the population in many countries. More vaccines are expected to be approved in the future. These vaccines use different technologies and have been developed in different ways. For instance, both the vaccine developed by the pharmaceutical company Pfizer together with the research centre BioNTech (henceforth, the Pfizer/BioNTech vaccine) and the one developed by the pharmaceutical company Moderna use a novel mRNA technology. The vaccine developed by the University of Oxford and produced by the pharmaceutical company AstraZeneca (henceforth, the Oxford/AstraZeneca vaccine) and the one produced by Johnson&Johnson use an adenovirus technique^1^. Like many other vaccines already widely used (e.g. against rubella, chickenpox, hepatitis A, and shingles), some of the current and likely of the future vaccines, including the Oxford/AstraZeneca and the Johnson&Johnson vaccines, have been developed by using cells that were replicated from HEK-293 cell lines obtained from foetuses after elective abortions. The abortions took place in the early 1970s, but as with most cell lines derived from aborted foetuses for research purposes in those years, little is known about the foetuses and the women who had the abortions (Wadman 2020a).

Both types of vaccines raise ethical issues around most appropriate vaccination policies, for example, about which groups to target first (Giubilini, Savulescu, Wilkinson 2020) and what level of coercion, if any, there should be (e.g. mandatory vaccination or some other measure). One ethical concern has been raised by representatives of certain religious groups who do not wish to receive COVID-19 vaccines that are linked to abortions (Wadman 2020b). The Catholic Archbishop of Sydney Anthony Fisher, for instance, wrote that “those who are troubled by [the COVID-19 vaccine] will either have to acquiesce to the social pressure to use the vaccine on themselves and their dependents, or conscientiously object to it” (Fisher 2020). Some of those who have previously defended a right to conscientious objection to vaccination in the name of religious freedom or freedom of conscience (e.g. Navin and Largent 2017) have applied their arguments to the future COVID-19 vaccines (Navin and Redinger 2020). According to these views, individual freedom, including religious freedom, should be guaranteed as long as it does not pose significant threats to the collective. Unlike some other vaccines, in the case of COVID-19 there are alternatives to a vaccine that uses fetal cell lines at the development stage —at the moment, in many countries, these alternatives are the Pfizer/BioNTech and the Moderna vaccines. While these vaccines have not been developed with the use of fetal cell lines, they have used those fetal cell lines at the testing phase. However, the connection with abortion is more remote than in the case of vaccines that use fetal cell lines at the development stage.

In a recent Note, the Congregation for the Doctrine of the Faith has stated that it can be morally permissible to use COVID-19 vaccines linked to abortion, given that the link to the abortion is remote and the use of the vaccine does not imply an endorsement of abortion. In theological language, using such vaccines is a form of “passive material cooperation.” According to the Note,

… all vaccinations recognized as clinically safe and effective can be used in good conscience with *the certain knowledge that the use of such vaccines does not constitute formal cooperation with the abortion* from which the cells used in production of the vaccines derive. (Congregation for the Doctrine of the Faith 2020, ¶3)

However, the Note also says that this applies only “when ethically irreproachable COVID-19 vaccines are not available” (¶2). This is precisely why, now that many different vaccines are available, the problem might become more relevant (Congregation for the Doctrine of the Faith 2020; Pontifical Academy for Life 2006 and 2017).

Thus, for instance, the U.S. Conference of Catholic Bishops urges that “to distance oneself as much as possible from the immoral act of another party such as abortion […], [t]he AstraZeneca vaccine should be avoided if there are alternatives available” and “the reasons to accept the new COVID-19 vaccines from Pfizer and Moderna are sufficiently serious to justify their use, despite their remote connection to morally compromised cell lines” (USCCB 2020, 5). In fact, the Pope himself received the Pfizer/BioNTech vaccine on January 12, 2021, and has emphasized the moral obligation for Catholics to get vaccinated against COVID-19 when eligible (Sly 2021).

Other vaccines currently being developed, such as those by GlaxoSmithKline and Sanofi Pasteur, would not make any use of fetal cell lines either at the development or at the testing phase, as far as we know at the moment. So, if they are approved, certain people would believe they have a moral obligation to use them and refuse the others.

Should countries that are providing COVID-19 vaccines to their populations take account of these concerns and allow people to choose alternatives that they do not find ethically problematic?

This ethical issue will become more relevant as soon as we have large enough availability of different vaccines and more people will have access to at least one of them.

Religious freedom, as part of a broader freedom of conscience, is an important value in liberal societies. However, we often need to strike a balance between it and other important values that secular societies consider at least as important, if not more important, especially in certain contexts like public health. These include fair and effective allocation of scarce public health resources and protection of public health and collective well-being.

In this paper we argue that requests to receive COVID-19 vaccines not linked to abortion on the basis of claims to religious freedom (and freedom of conscience more broadly) should be accommodated only on the following conditions:

– There is sufficient availability of the alternative vaccines such that the choice does not prevent other people in a target group (whichever it is) from getting the best vaccine for that group (whichever it is). In other words, giving objectors an alternative vaccine should not undermine whatever public health strategy is in place.
– The person requesting the alternative vaccine pays for any significant additional cost of such a vaccine, so that the objection does not impose additional costs on the community.
– The same option is offered to people who object to vaccines for other moral reasons, whether related to some other religious view or secular view.

In practice, satisfying all these conditions at the same time might turn out to be very difficult.

## Relative Advantages and Disadvantages of Current COVID-19 Vaccines

The COVID-19 vaccines we currently have and will likely have in the future differ from each other in several ways, which are relevant from a public health and an ethical perspective. We describe here some of these differences, which are also summarized in Table 1 above.

The Pfizer/BioNTech and Moderna vaccines have shown very promising results in terms of safety and effectiveness, at least at preventing serious symptoms and deaths. The estimate is that, after 90 days from the second dose, the Pfizer/BioNTech vaccine is 78 per cent effective at preventing high viral loads from the Delta variant (Powels et al. 2021) Importantly, these vaccines have shown high effectiveness on old age groups. Thus, many countries are distributing them according to a largely age-based priority order (adjusted to include some frontline workers among the high priority groups) to ensure that the most vulnerable will be the first to be immunized. This applied to the first dose of the vaccine and now applies to the third so-called “booster dose,” which some consider necessary in light of the quickly waning immunity conferred by the first two doses. The Oxford/AstraZeneca also proved to be very safe but showed lower effectiveness, though with mixed results. Clinical trials showed around 70 per cent effectiveness across all age groups but with some interesting variation on the basis of dosage, with 90 per cent effectiveness if administered through a half dose followed by a full dose and 62 per cent effectiveness if administered through two full doses. It is unclear at the moment whether the more effective dosage would work equally well across different age groups, as it was only observed in a group younger than 55 (Ramasamy et al. 2020; Voysey 2020; Knoll and Wonodi 2021). Against the Delta variant, the Oxford/AstraZeneca vaccine is estimated to be 61 per cent effective against high viral load (Pouwels et al. 2021)—that is against severely symptomatic cases that are more likely to result in hospitalizations and deaths. Clinical trials suggest that the Johnson&Johnson vaccine is 66 per cent effective at preventing symptomatic infections. Like the Oxford/AstraZeneca, the Johnson&Johnson vaccine has very high effectiveness at preventing severe cases and hospitalizations (FDA 2021).

**Table 1:**
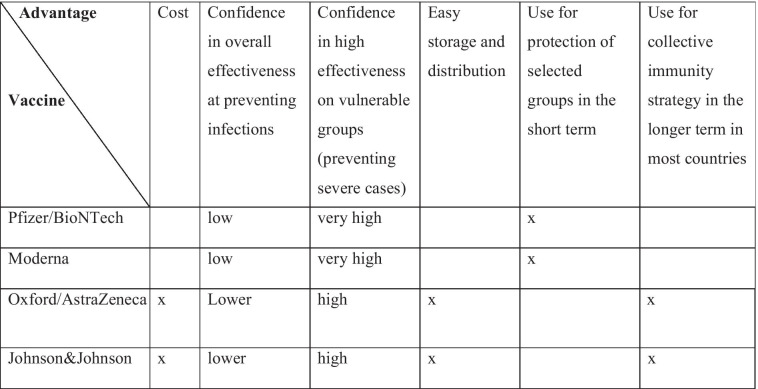
Comparison of different advantages of current COVID-19 vaccines

However, it is also important to point out that all these vaccines’ effectiveness at preventing infection and transmission against the Delta variant is likely to be significantly lower, though there is a lot of uncertainty around this aspect. According to a study by Imperial College, London, double-vaccinated people have between 50 per cent and 60 per cent reduced risk of infection (Elliot et al. 2021). However, if they do get infected, CDC data indicate that with the Delta variant the viral load of vaccinated people is roughly the same as that of infected unvaccinated people (Brown et al. 2021).

All these different characteristics might in the future justify different vaccination policies with regard to the different types of vaccines. For example, when availability increases and the most vulnerable have been vaccinated, one option for mass vaccination might be to distribute the Oxford/AstraZeneca or the Johnson&Johnson vaccine (or other similar ones that will be approved) among younger population groups and the Moderna and Pfizer/BioNTech ones (or other similar) among older groups, given the latter’s higher effectiveness in older age group (who need the vaccine the most) but also their larger cost.

Indeed, one important difference between vaccines is in their cost. The Pfizer/BioNTech and Moderna vaccines are quite expensive (£29.26 for the two doses of the Pfizer/BioNTech one, and £37.5 − £55.52 for the two doses of the Moderna one), and their distribution is made difficult by the fact that they need to be stored at very low temperatures (-70C for the Pfizer one, and -20C for the Moderna one, though after thawing the Moderna one can be preserved at normal fridge temperature for thirty days) (Brown 2020)—although some countries are enhancing their storage and distribution capacities^2^. In a country like the United Kingdom, this means that GP practices and pharmacies, where vaccinations are normally administered, have limited capacity to store and provide these vaccines. Both aspects would make it very difficult and often practically impossible to use such vaccines in low-income countries or to rely completely on them even in high-income countries. Even if it is true that countries like Israel or the United States have successfully mass-vaccinated using the Pfizer/BioNTech vaccine, many countries would either not be able to afford such costs or, quite reasonably, prefer to rely significantly on less expensive vaccines for mass vaccination. This will be even more likely given that Pfizer is now increasing the cost of its vaccine significantly, to US $23 per dose in the new contracts being signed with the European Union (Reuters 2021). When it comes to mass vaccination, many countries—especially low and middle income ones—will want or need to rely to a significant extent on cheaper and easier to distribute vaccines such as the Oxford/AstraZeneca one, the Johnson&Johnson one, or any future vaccine that shares their features. The Oxford/AstraZeneca vaccine does not need very low storage temperature and, very importantly, is much cheaper (approximately £4.50 if the vaccine is used with the half/full dose regime). The same could be said for Johnson&Johnson’s vaccine, which costs around US $10 per dose, but that only requires one dose, making it not only economically but also practically more feasible. These cheaper vaccines might well be the only way out of the emergency for low- and middle-income countries and an essential part of the way out for high-income countries as well.

Thus, all in all, the Oxford/AstraZeneca vaccine and the Johnson&Johnson vaccines have advantages over the Pfizer/BioNTech and Moderna vaccines in terms of potential for large scale distribution, especially in low and middle-income countries, and for vaccination strategies aimed at immunizing large portions of the population. Vaccination strategies will need to take these advantages into account. For example, when it comes to mass vaccination in the long term, especially if vaccines will be required on a regular basis to prevent immunity from waning, a country might be able to offer the cheap Oxford/AstraZeneca or Johnson&Johnson vaccine completely free of charge but only partially subsidize for certain groups an expensive vaccine like the Pfizer/BioNTech or Moderna ones. Or a country might choose strategies that rely more heavily or exclusively on cheaper vaccines because of financial constraints.

Once we consider all these factors, it becomes clear that giving people a completely free choice of the vaccine might not be without public health and economic costs. Religious freedom in this case could stand in the way of important public health goals or the public goods we want to achieve through vaccination policies in the most cost-effective way.

Of course, other factors might determine what at any one time is optimal vaccine roll out. An obvious one is the risk profile. At the time of writing, some countries (including the United States, the United Kingdom, and European Union countries) have either suspended or limited the use of the Oxford/AstraZeneca and the Johnson&Johnson’s vaccine after a link was found with very rare cases of blood clots. Whether the risk assessment in these cases has been appropriate is an issue that would deserve a separate discussion. However, since, as a matter of fact, even minuscule risks of vaccines are affecting vaccination strategies, it would also be relevant to consider how the free choice of the vaccine would affect vaccination strategies in terms of fair distribution of vaccine risks.

As we shall see, the problem becomes even more marked if we consider that the kind of freedom advocated cannot be applied only to specific religious views, which would be an unjustified form of religious privilege. In secular societies, the same kind of freedom, if granted to one specific religious views, should consistently be applied to other views around vaccines, whether religious or secular. The risk of compromising vaccination strategies would then be higher.

## Vaccination Strategies and Religious Freedom

An ethical conflict arises if an individual is eligible to receive a publicly funded vaccine to which they have moral objections (for example, a conservative Catholic person who is eligible to receive the Oxford/AstraZeneca vaccine). Should the State pay for the more expensive alternative?

It is useful to consider the same ethical problem when it arises in the context of individual clinical decisions and then compare it with the case of public health decisions.

One principle that publicly funded healthcare systems might draw on is the following:

The Optimal Treatment Principle: For a given condition, publicly-funded healthcare systems should provide only the most effective treatment that is both available and affordable. (Wilkinson and Savulescu 2018, 290)

According to this principle, public healthcare systems should not provide suboptimal treatment. A treatment can be said to be suboptimal for an individual when it has one or more of the following, compared to available alternatives: reduced magnitude of benefit; reduced probability of benefit; reduced duration of benefit; increased magnitude of harm; increased probability of harm; reduced cost-effectiveness; or, reduced evidence about actual costs and risk/benefit ratio (Wilkinson and Savulescu 2018).

The Optimal Treatment Principle can be justified on the basis of considerations of beneficence, non-maleficence, and a reasonable conception of justice. Acting in the patient’s best interest requires maximizing the health benefit of the scarce health resources we are using for them. Making the most of a limited resource is also a matter of justice both from a contractualist and a utilitarian perspective. Giving limited health resources to those who could benefit the most from them and in such a way that would minimize further costs on the collective seems something that everyone would accept from “behind a veil of ignorance” (for instance, ignorance with regard to one’s own future religious views) and that would maximize both the individual and collective good. There can be reasonable disagreement around what counts as “benefitting the most,” as some people might reasonably think that the benefit is defined by reduced chances of dying from a certain condition, by life of years saved, by the expected quality of the remaining life, and possibly other aspects. But most would agree that these are the kinds of considerations that should drive allocation of scarce medical resources.

A missing ethical principle here is, of course, autonomy, which is often considered the most important principle in contemporary biomedical ethics. It is normally considered acceptable for competent patients to refuse treatments or to request suboptimal treatments compared to an available alternative. The choice might be based on judgements about whether they would medically benefit from the intervention or whether the side effects of treatments are worth their benefits. However, it might also be based on ethical or religious views. One of the textbook examples is that of the Jehovah’s Witness person who is entitled to refuse blood transfusions for themselves even when they might be life-saving. A principle of religious freedom in medical ethics can be defended on the basis of a more general principle of autonomy.

In this paper we focus on religious freedom because it is the specific principle that is being invoked with regard to the link between vaccines and fetal cell lines (e.g. Navin and Redinger 2020). However, as we are going to mention below, nothing in what we say here suggests that our considerations are limited to religious opposition to certain vaccines. What is really at stake here is a more general principle of autonomy, or of freedom of conscience, of which religious freedom is a particular instantiation.

However, autonomy is not an absolute principle and needs to be balanced against other considerations, most notably fairness in allocation of scarce healthcare resources. Fairness requires that treatments provided are not only effective but at least to a certain degree cost-effective, since we want to make the best use of finite resources so as to free up as many of them as possible for others who need them. This requirement also implies an ethical duty to minimize future healthcare expenditures that the failure to provide the optimal treatment would entail.

Balancing principles implies that there is a limit to the cost we should be prepared to pay to respect autonomy (including religious freedom). Once the cost becomes too large in terms of sacrifice of other important values, autonomy may permissibly be limited—though of course people disagree on what counts as “too large.” Thus, in the medical context, if a suboptimal treatment results in requests for additional healthcare resources that the optimal treatment would likely have prevented or contained, it is reasonable to expect those requesting the suboptimal treatment to cover such costs, at least when they are beyond a certain limit. This strikes a reasonable balance between autonomy and fair allocation of scarce resources (Wilkinson and Savulescu 2018).

Similar considerations can be made with regard to public health interventions, although the definition of a suboptimal intervention in this area is slightly different. Analogously to the case of individual clinical intervention, a public health policy is suboptimal if it reduces magnitude, probability, or duration of benefit for the collective or increases magnitude or probability of harm for the collective, or is less cost-effective or has reduced evidence about costs and harm/benefit ratio, or any combination of these factors. However, public health interventions typically need to strike a different type of balance between individual and collective interest than individual medical interventions.

In the case of individual medical interventions, the intervention is optimal if the best outcome for the individual is achieved while minimizing the negative impact on the collective (most notably through unfair use of scarce resources). A public health intervention is optimal if the best outcome for the collective is achieved while minimizing financial or other types of costs for the collective and for single individuals.

It is the same problem that, absent vaccines, arises with regard to other pandemic measures. A public health intervention like a lockdown, for example, is suboptimal if the costs it imposes on individuals (for instance, physical or mental health impact of social isolation) are too large and not worth the collective benefit. Identifying what kind of intervention is suboptimal in public health when individual and collective interests are in tension in such an extreme way can be difficult. However, the individual cost of vaccination is very small (assuming vaccines’ relative safety) and the intervention is actually beneficial (assuming vaccines’ high effectiveness). Thus, it is very unlikely that burdens of vaccination on individuals would make vaccination policies suboptimal, in the way in which lockdown policies might be.

Thus, protecting the religious freedom and, more generally, the autonomy of those who have moral objections to a vaccine should not pose significant costs in terms of hindering public health goals and in terms of costs for healthcare systems. Public health strategies are a context where autonomy and religious freedom are of secondary importance compared to the public health considerations that ground such strategies. Religious freedom cannot be expected to have in public health policy the same special place that it is normally bestowed in contexts where promoting freedom and pluralism is a primary goal (such as policies on freedom of speech or freedom of association). After all, this idea has been extensively applied in the case of recent pandemic measures, where individual liberties have been largely sacrificed to protect the most vulnerable to COVID-19 and healthcare systems, including restrictions on religious freedoms (such as on attendance at Mass or other religious ceremonies).

It is true that religious freedom has been protected in the United States more than in the European Union, for example in the case of the Supreme Court blocking some limitations on religious services introduced by some U.S. states during this pandemic. However, not all of them have been blocked. And, more generally, the priority of religious freedom over public health concerns started to be questioned in the United States even before the pandemic. For example, New York eliminated religious exemptions to the MMR vaccine requirement for school enrolment in 2019 to tackle the problem of frequent measles outbreak, and at the moment five states allow no non-medical exemption to school vaccination mandates on the basis of religious or personal beliefs (NCSL 2021). Given that COVID-19 is posing a larger risk to public health than measles (at least judging from the response to it), it would not be too surprising if the prioritization of public health over religious freedom would become central in COVID-19 vaccination policies as well.

What does this mean in practice?

If the different COVID-19 vaccines available were roughly equally expensive, equally accessible, and equally effective on the same population groups, then people who are eligible to receive a COVID-19 vaccine should be left free to decide which one to receive on the basis of personal moral or religious views without any additional cost. This is because autonomy and religious freedom matter. *Other things being equal*, individual autonomy, and therefore religious freedom, should be respected.

However, the problem arises when other things are not equal. Respect for autonomy and religious freedom in vaccination policies can pose costs on the collective or on particular individuals. These are either significant financial costs (e.g. because certain vaccines are more expensive) or costs in terms of negative impact on public health strategies (e.g. by slowing down the delivery of the right vaccine to the right groups of people). In a moment when time is of essence in order to preserve lives, any delay implied by policies intended to accommodate objections to specific vaccines might translate into more lives lost.

We would need to make sure that such cost is borne by those who claim such freedom, rather than on others. This is because the aforementioned public health ethics constraints apply, including fairness constraints. Fairness might require objectors to internalize the costs even if the number of people requesting alternative suboptimal vaccines is small and therefore their impact on public health strategies is minimal. (One obvious objection is that we might be able to accommodate a small number of claims to free choice without significant collective cost. We will address this at the end of the article.) If there was a fundamental right a stake, or even a “human right”, the cost of respecting that right should arguably be borne (to a significant extent at least) by the collective, to make sure differences in wealth do not affect the extent to which individuals enjoy their basic rights. However, the point we are making here is precisely that being allowed to choose one’s ethically preferred vaccine should not be seen as a fundamental individual right.

At the moment, the vaccines not linked to abortion are significantly more expensive than those linked to abortion, as we saw above. A healthcare system should only subsidize the cheapest and most effective option in order to fulfil its obligation to protect public health, making the most efficient use of scarce resources.

Allowing choice of the vaccine but making it conditional upon individuals paying any cost difference would represent a reasonable accommodation of religious freedom. It might be an acceptable compromise if there is enough availability of different vaccines. However, this may not be possible if vaccine availability is extremely limited, such that providing choice of vaccines deprives other people of the most appropriate vaccine (or of a vaccine at all). For example, if there is insufficient mRNA vaccine to provide to higher risk groups, it may not be appropriate to allow lower risk patients to choose that option. In such cases, it may not be appropriate to accommodate religious freedom, given the importance in public health ethics during a pandemic of securing most protection for the vulnerable. When there is highly limited supply, patients should only be offered the vaccine that is most indicated for their group (or, if vaccination is made mandatory, they should be subject to whatever requirement is in place for that vaccine).

## Religious and Secular Objections Should be Treated Equally

Religious beliefs in liberal secular societies should not be privileged compared to secular moral beliefs. This reflects the more general point that if religious values are accommodated in healthcare choices, other non-religious requests must be treated in the same way in order to avoid religion-based discrimination (Savulescu 1988). Refusal to vaccinate or refusal to receive certain types of vaccines can be motivated by different types of beliefs, either factual (e.g. beliefs about risks of the vaccine) or ethical (e.g. beliefs in natural lifestyles and natural medications). Refusal based on risk perception can also reflect a type of ethical assessment.

For example, someone might prefer an mRNA vaccine to an adenovirus vaccine because they believe (possibly erroneously) that it is safer or more effective. It is true that risk assessment is based on factual information, but whether risks are worth taking for any individual is a value choice and often a moral choice. For instance, someone with dependents might think that it would be irresponsible for them to opt for the unknown risks of long-term side effects of the mRNA vaccine compared to the risks of an adenovirus vaccine, given their caring responsibilities. Or someone might feel more responsible if they suffered injuries from the vaccine which they intentionally decided to take, as opposed to getting sick naturally and unintentionally by getting infected from COVID-19—after all, this kind of omission bias has been observed in other vaccination decisions (DiBonaventura and Chapman 2008; Ritov and Baron 1992; Asch et al. 1994). The fact that it is considered a bias does not detract from the respect owed to it *qua* personal belief, any more than the non-evidence base of religious beliefs does. From the point of view of public ethics, the two stand or fall together.

Granted, not all of these secular views might have the same status as religious views. It could be suggested that religious views are typically part of comprehensive worldviews, while secular beliefs around, say, the risk assessment on mRNA vaccines are more often specific concerns not related to broader worldviews. This might be taken to make a difference to the respect owed to each of these views when it comes to public policy. Even accepting this (not implausible) view, it is still the case that many secular beliefs around vaccines can be part of comprehensive worldviews, for example about the importance of natural lifestyles, the legitimate use of animals in research, and so on. Thus, even if our point does not apply to all secular beliefs about vaccines, it does apply to many of them.

If we allow religious objectors to access the mRNA vaccine, we should allow many others to access it for personal reasons. And if accessing more expensive alternatives requires internalizing the cost of the choice, this should apply to religious and non-religious requests alike.

However, this approach would also increase the risk of jeopardizing overall public health vaccination strategies because more people would claim such liberty.

If those who refuse a specific vaccine for *any* reason are too many and they risk compromising public health strategies, then it may be justified not to offer vaccine choice. This should apply to both religious and non-religious requests.

If those who refuse a certain COVID-19 vaccinations for *any* reason would not compromise public health strategies, then there would be no need to restrict vaccination choice for anyone, regardless of whether the objection is religious in nature. However, once again, this would likely entail some costs if the morally preferred vaccine is more expensive. For the reasons explained above, those making this choice should internalize such costs.

Requiring people to internalize the cost of their choice is a mild limitation of individual liberty because not everyone might afford or might be willing to pay the cost. If we adopt it, then the same kind of limitation should apply to religious and non-religious objections alike. The degree of liberty that people enjoy in liberal, secular societies should not depend on whether people hold certain types of religious views.

## Meeting Two Objections

One potential concern is that not offering the choice of a vaccine (particularly where there are ethical or religious reasons that are important to people) might backfire by reducing people’s willingness to vaccinate. It might lead to negative publicity around vaccination and risk worse overall public health. If our worry is that giving a suboptimal vaccine might compromise public health strategies, surely a suboptimal vaccine is still better than no vaccine at all. This consideration is more relevant in a context where vaccination is not mandatory, and therefore vaccine refusal may lead to a failure to achieve adequate levels of collective immunity. (Whether vaccine mandates would instead be effective is beyond the scope of this article).

This is a reasonable concern. However, we also need to consider the possibility, raised in the previous section, that providing choice of vaccine will undermine strategies of vaccine distribution. If we make the choice available to anyone irrespective of their moral or religious views (and there is a restricted supply of vaccines), then this risk is real. Thus, vaccination strategies could be compromised either way. We should not simply assume that a higher collective uptake with a large proportion of suboptimal individual vaccinations would be better than a lower collective uptake where all those who do get vaccinated receive the best vaccine for them.

These issues largely come down to empirical considerations and expectations about people’s behaviour, more than to ethical considerations. It is hard to tell whether the risk of restrictive policies backfiring is larger than the risk of too-liberal policies backfiring.

A second objection is that we might be able to accommodate at least a small number of claims to free choice of the vaccine without compromising public health strategies or posing significant costs on the collective. If we can do that, surely, we should, as we would achieve the same collective benefit with less liberty infringements. This might be a better way of balancing fairness, collective good, and individual freedom. However, we would need to consider whether the mechanism used to identify those allowed to object is in itself fair. For instance, a first come/first served basis for granting free choice of the vaccine is unlikely to be a fair system. Those who come first are likely to be either those who have priority access to the vaccine or easier access to healthcare services. The fairer system might be a lottery among all those who will be vaccinated in the short and long term and who would want the free choice. A lottery might be quite difficult to implement and to consistently keep in place over time. It is also worth mentioning that it is not a solution normally adopted in other contexts where a small number of outliers would not “make a difference.” For instance, we do not have a lottery to allow a small number of individuals to decide what their taxes should and should not fund, even if we can certainly afford a certain number of such individuals. This suggests that either a lottery solution is not very easily implementable or that we think that it would still be unfair towards those who do make their fair contribution. One possibility is that the unfairness in question lies not so much in the procedure to determine whom to exempt but in the idea that we should have exemptions in the first place.

We are however happy to concede that the lottery model is an option worth considering if we agree it is fair and accommodates individual liberties at no significant collective cost.

## Conclusion

Distributing COVID-19 vaccines in the most effective way is the most urgent goal and the primary responsibility of governments in designing vaccination strategies. There is also a strong ethical requirement to make the distribution cost-effective, at least as long as availability is limited (but probably also after this phase). These requirements imply that vaccination strategies should be guided primarily by considerations around public health and effective use of limited resources.

Different vaccines’ characteristics might require that different vaccines be distributed in different ways and to different groups in the pursuit of such goals. Whether vaccines have been obtained with the use of aborted foetuses is not a consideration that is relevant to the pursuit of such strategies, and protecting religious freedom is not a priority of public health interventions (to say that it is not a “priority” does not mean that it is not important).

However, as long as public health interventions are carried out in liberal societies, individual freedoms should be given some consideration, in a way that does not significantly affect public health priorities.

Thus, if there is sufficient availability of different vaccines and giving people the choice of which vaccine to receive on the basis personal religious beliefs does not compromise public health goals, then people should be allowed to choose which vaccines to receive. However, they should pay for any additional cost of this choice. This should extend to non-religious requests for vaccine alternatives.

Limited availability of the vaccine might still make a small number of exemptions affordable. If so, we might want to consider the very unusual solution of a lottery to determine who will enjoy the privilege of the free choice, but we would need more discussion to determine whether this would be fair.

If we do not want to consider the lottery solution and if there is no sufficient availability to allow this qualified form of religious freedom, then people should be subject to whichever vaccination requirement is in place or have access only to whichever vaccine is targeted to their group.

## Footnotes

1 In this article we focus on these four vaccines for the simple reason that at the moment they are the most widely used, or most likely to be widely used, in the foreseeable future in most Western countries, and they exemplify well the ethical and religious concerns here discussed. The same points we make here could be applied to any current or future approved vaccine which shares the same problematic features.

2 See e.g. Australian Department of Health. 2021. Wider storage and transportation conditions for the Pfizer COVID-19 vaccine now approved, April 8. https://www.tga.gov.au/media-release/wider-storage-and-transportation-conditions-pfizer-covid-19-vaccine-now-approved. Accessed October 20, 2021.
